# The Therapeutic Effect of Berberine in the Treatment of Nonalcoholic Fatty Liver Disease: A Meta-Analysis

**DOI:** 10.1155/2016/3593951

**Published:** 2016-07-03

**Authors:** Xiaoyun Wei, Chunyan Wang, Shijun Hao, Haiyan Song, Lili Yang

**Affiliations:** ^1^Institute of Digestive Diseases, Longhua Hospital, Shanghai University of Traditional Chinese Medicine, Shanghai 200032, China; ^2^China-Canada Center of Research for Digestive Diseases (ccCRDD), Shanghai 200032, China

## Abstract

*Aim*. To assess the efficacy of berberine in the treatment of nonalcoholic fatty liver disease through meta-analysis.* Method*. We searched Embase, Pubmed, Cochrane Library, and so forth, until March 2016 for randomized controlled trials using berberine to treat NAFLD.* Result*. Six randomized controlled trials involving 501 patients were included in this study. The results showed that the efficacy of reducing TC, LDL, ALT, 2hPG, and HbA1c in NAFLD patients of the berberine group were significantly higher than that of control group. The subgroup analyses on TG, AST, and FBG indicated that treatment combined with berberine decreased TG level in NAFLD patients significantly. Compared with other drugs, berberine alone decreased TG level in NAFLD patients significantly. We also conducted a descriptive analysis on insulin resistance and radiography results that berberine can improve NAFLD patients' insulin resistance and fatty liver.* Conclusion*. According to analysis result, berberine has positive efficacy on blood lipids, blood glucose, liver function, insulin resistance, and fatty liver condition of NAFLD patients. However, due to the limitation of number and quality of trials included, more clinical randomized controlled trials with high quality are needed for further verification of the efficacy of berberine on NAFLD patients.

## 1. Introduction

Nonalcoholic fatty liver disease (NAFLD) is a kind of metabolic stress-induced liver injury which is closely related to insulin resistance and genetic susceptibility. The spectrum of this liver disease includes nonalcoholic simple fatty liver (NAFL), nonalcoholic steatohepatitis (NASH), relevant liver cirrhosis, and hepatocellular carcinoma [1]. Around the world, the morbidity of NAFLD is 6.3%–33% [2–4], while the morbidity in overweight people and type 2 diabetic patient is, respectively, up to 58% and 74%. One longitudinal analysis of clinical data indicated that about 1/3 of NAFL patients could develop into NASH, and once they developed into NASH, the risks of liver cirrhosis, hepatocellular carcinoma, and liver failure may increase markedly [5]. Finally, these risks may lead to liver disease related disability or death of patients.

The pathogenesis of NAFLD has not been completely clarified yet. Nowadays, the possible pathogenesis involves two-hit hypothesis, insulin resistance, leptin resistance, oxidative stress, endoplasmic reticulum stress, and alteration of intestinal flora. The main therapies of this disease in modern medicine are change of life style intervention and drug therapy. In recent years, researches aimed at new drugs to treat NAFLD have not achieved new breakthrough, and the most used drugs are metformin and thiazolidinediones (in order to improve insulin resistance) as well as statins (in order to reduce blood fat). Therefore, to research and develop an effective drug which is effective in NAFLD is very significant to present medical situation.

Berberine, also called berberine hydrochloride, is the main active ingredient of traditional Chinese medicines Coptis Root and Cortex Phellodendri. Berberine is a common kind of isoquinoline alkaloid whose molecular formula is C_20_H_18_NO_4_. For the past few years, extensive researches found that berberine could regulate blood glucose level, reduce blood lipid, provide an effect of antiarrhythmia and antiplatelet aggregation, enhance body immunologic function, and so on [6–9]. The latest researches demonstrated that berberine was a quite good effective drug to treat NAFLD. The pharmacokinetics research shows that liver is the organ which contains the highest concentration of berberine metabolites, and the concentration in liver is about 70 times as large as that in plasma [10]. In addition, the half-life of berberine in liver tissue is longer than that in other tissues, and these results may explain that liver is the main target organ of berberine [11]. In the study of Li et al. [12], berberine can promote the excretion of cholesterol from liver to bile and, as a result, blood lipid can be reduced. Furthermore, berberine can regulate lipid metabolism and improve hepatic steatosis through increasing the expression of low density lipoprotein receptor (LDLR) [13]. In the study of Pérez-Rubio et al. [14], berberine can enhance patients' insulin sensitivity through PPAR-*γ* pathway and promote tyrosine phosphorylation in insulin receptor substrate (IRS); thus insulin resistance can be improved. Meanwhile, berberine can increase the amount of glucose transporter 4 (Glut-4) which can accelerate the ingestion of glucose and, as a result, blood glucose can be reduced [15].

Though there are several clinical trials to verify the preferable efficacy and toxic and side effects of berberine on treating NAFLD, the effectiveness of berberine has not been proved precisely due to small sample size. This study aimed to evaluate the therapeutic effect of berberine in treating NAFLD using meta-analysis in order to provide evidence for clinical decision.

## 2. Methods

### 2.1. Data Sources and Searches

**Figure 1:**
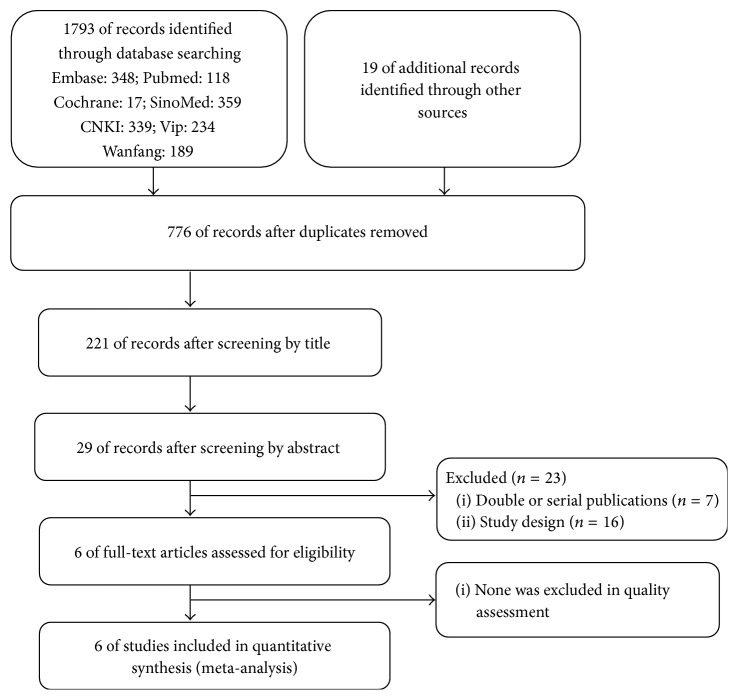
Identification of included studies.

The article selection process is shown in Figure 1. Electronic searches up to March 2016 were conducted in Embase, Pubmed, Cochrane CENTRAL Register of Controlled Trials, and Cochrane Database of Systematic Reviews. With regard to Chinese databases, SinoMed, Chinese journal full-text database (CNKI), VIP database, and Wanfang digital periodical full-text database were searched. The references lists of articles identified in the electronic search were hand-searched for other relevant articles.

Search terms were NAFLD, nonalcoholic fatty liver disease, NASH, nonalcoholic steatohepatitis, fatty liver, fatty liver disease, randomized controlled trial, controlled clinical trial, berberine, BBR, and huang lian su.

### 2.2. Study Selection

Inclusion criteria were English and Chinese articles with participants who are aged older than 18 years, who are of any sex or ethnic origin with NAFLD, who conformed to the diagnostic criteria of NAFLD (like China, the United States, Japan, Italy, and other guidelines).

Exclusion criteria were nonhuman studies, drug-induced, total parenteral nutrition-induced, and viral or genetic causes of liver injury, nonrandomized controlled trials, studies enrolling fewer than 10 subjects, case reports, reviews, and treatment time of the studies less than 2 weeks.

### 2.3. Intervention Measures

Berberine group (experiment group) was versus placebo group, lifestyle intervention (ISI) group, or other medicines' group (control group). Berberine group combined with other medicine group (experiment group) versus corresponding medicine group (control group).

### 2.4. Outcome Indicators

Total cholesterol (TC), triglyceride (TG), low density lipoprotein (LDL-C), high density lipoprotein (HDL-C), alanine transaminase (ALT), aspartate transaminase (AST), gamma-glutamyl transferase (GGT), fasting plasma glucose (FPG), 2 h postprandial plasma glucose (2hPG), glycated hemoglobin (HbA1c), insulin resistance (IR), and hepatic pathology.

### 2.5. Data Extraction, Management, and Analysis

Two authors independently extracted data from all included articles (Lili Yang and Xiaoyun Wei), and any disagreement was discussed and documented. Again, a third author settled disagreements that could not be solved by discussion.

Authors of studies were contacted for clarification when necessary. The quality of randomized controlled trials (RCTs) was assessed by the Cochrane Risk of Bias Tool, attributing 1 point to each item.

Review Manager (RevMan 5.3) was used to estimate pooled mean difference (MD) for continuous outcomes and odds ratio (OR) for binary outcome measures. 95% confidence interval (95% CI) will be used as effective size for the combined analysis. Heterogeneities were estimated using the *I* ^2^ statistics. When *I* ^2^ < 50% and *P* > 0.10, the results were considered homogeneous and the fixed-effect model was used; when 50% ≤ *I* ^2^ < 75%, the results were considered heterogeneous and the random-effect model was used. When *I* ^2^ ≥ 75%, sensitivity analysis or subgroup analysis was conducted to identify the causes of the heterogeneity, and if *I* ^2^ remained 75% or greater, we only provided descriptive results without pooling estimates. Statistical significance was set at *P* < 0.05. A funnel plot was used to evaluate publication bias.

## 3. Result

### 3.1. Study Description and Quality Assessment

Initially, we totally searched out 1812 records and then retained 776 records after removing duplicate records. Excluding animal experiments, case reports, reviews, and articles with incongruent intervention measure and research orientation, we achieved 6 records: 1 in English [21] and 5 in Chinese [16–20]. Among these records, the study of Yan et al. 2015 [21] includes two control groups: ISI group and pioglitazone group, so we divided this study into Yan et al. 1.2015 and Yan et al. 2.2015, two parts, before analysis. The characteristics of studies included are clarified in Table 1, the publication year ranges from 2011 to 2015, and the case load ranges from 44 to 155 (median is 84). Total case load is 501 and, among them, 231 patients are in treatment group and 270 patients are in control group.

For all the studies included, we developed an analysis of patients' baseline information. The result shows that baselines of patients' data are not different between two groups, and all of the studies meet the inclusion criteria with good compatibility. The quality assessment of studies included is performed in Table 2. In the aspect of random sequence generation, 1 study applied random number table to group [18]; 1 study applied random allocation sequence produced by computer [21]; 4 studies mentioned “random” but without details of method. In the aspect of blinding, 1 study mentioned blinding but without details of method; 5 studies did not report situation of blinding and all the studies included did not report the plan of allocation and concealment. Four studies had no report about the withdrawals but provided complete outcome data, while the other 2 studies, respectively, had 1 [17] and 29 [21] patients withdrawal with the cause of loss to follow-up.

### 3.2. The Effect of Berberine on Blood Lipids in NAFLD Patients

**Figure 2:**
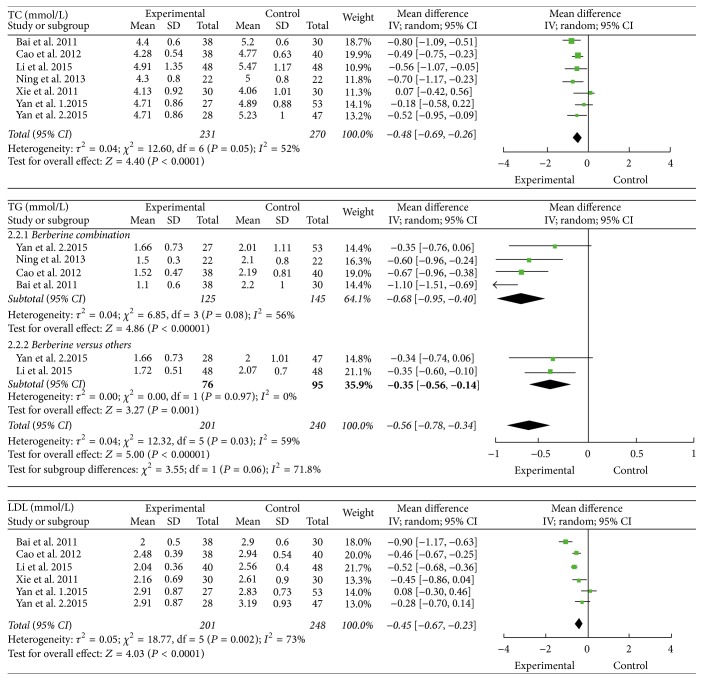
The effect of berberine on blood lipid in NAFLD patients.

Six trials reported the data of TC and TG. These trials involved 501 patients, with 231 patients and 270 patients in treatment groups and control groups, respectively. There was heterogeneity (*I* ^2^ = 52%) among these trials for TC. We conducted a random-effects model for TC, the result indicated that the reduction of the levels of TC in NAFLD patients who received berberine was more obviously than ones who received other drugs or lifestyle intervention (TC mmol/L: MD = −0.52; 95% CI −0.95 to −0.09; *P* < 0.0001). There was high statistical heterogeneity for TG (*I* ^2^ = 81%, *P* < 0.0001). Therefore, we performed subgroup analysis according to berberine alone groups and berberine combination groups, including berberine combined with lifestyle intervention or other drugs. A random-effects model analysis indicated that the combination with berberine significantly reduced the levels of TG in NAFLD patients (TG mmol/L: MD = −0.68; 95% CI −0.95 to −0.40; *P* < 0.0001). There was high heterogeneity (*I* ^2^ = 79%) among trials when comparing berberine alone to other drugs. Sensitivity analysis suggested that the study carried out by Xie et al. 2011 [20] made a great contribution to this high heterogeneity. When this study was excluded, the statistical between-studies heterogeneity (*I* ^2^) was 0%, *P* = 0.97. Further analysis manifested that Xuezhikang, the hypolipidemic medicine commonly used in clinic in [20], may be the main factor for the heterogeneity. So this study was excluded; as a result we found that berberine alone could significantly reduce TG than other drugs in the NAFLD patients (TG mmol/L: MD = −0.35; 95% CI −0.56 to −0.14; *P* < 0.0001) (Figure 2).

Five trials reported the data of LDL. These trials involved 449 patients, with 201 patients and 248 patients in treatment groups and control groups, respectively. The result showed statistically significant heterogeneity among studies with *I* ^2^ of 73%, *P* = 0.002. The result of a random-effects model analysis showed that berberine significantly reduced the level of LDL in NAFLD patients when compared with other drugs or lifestyle intervention (LDL mmol/L: MD = −0.45; 95% CI −0.67 to −0.23; *P* < 0.0001) (Figure 2).

Four trials reported the data of HDL. These trials involved 389 patients, with 171 patients and 218 patients in treatment groups and control groups, respectively. There was a substantial heterogeneity between trials with *I* ^2^ of 88% (*P* < 0.00001). Though sensitivity analysis and subgroup analysis were performed, the high heterogeneity remained. Therefore, we only did descriptive analysis. Two of these trials [17, 18] reported that berberine increased the level of HDL significantly in NAFLD patients. And the other two trials [20, 21] reported that berberine was likely to increase the level of HDL in NAFLD patients (Figure 2).

### 3.3. The Effect of Berberine on Liver Function in NAFLD Patients

**Figure 3:**
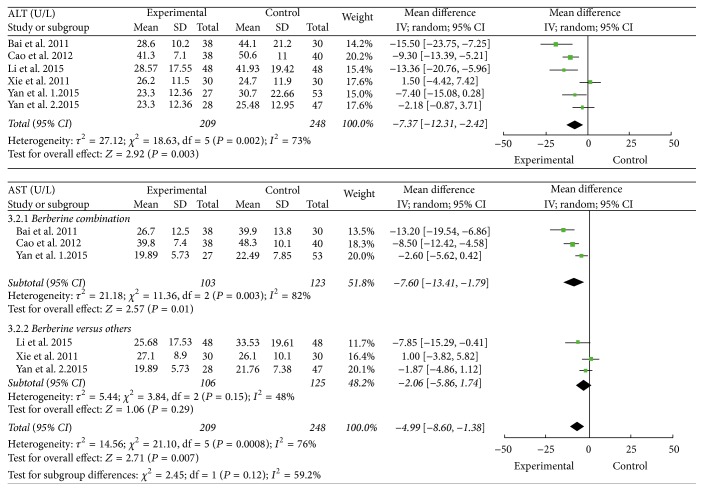
The effect of berberine on liver function in NAFLD patients.

Five trials reported the data of ALT and AST. These trials involved 457 patients, with 209 patients and 248 patients in treatment groups and control groups, respectively. There was a heterogeneity for ALT with *I* ^2^ of 73%. A random-effects model demonstrated that the reduction of ALT in NAFLD patients treated by berberine was more significant than that of other drugs or lifestyle intervention (ALT U/L: MD = −7.37; 95% CI −12.31 to −2.42). There was high statistical heterogeneity for AST (*I* ^2^ = 76%, *P* < 0.0001). The subgroup analysis was conducted according to berberine-alone groups and berberine combination groups. It revealed that the berberine-alone groups were more likely to reduce AST in NAFLD patients than other drugs or lifestyle interventions, which were analyzed by a random-effects model (AST U/L: MD = −2.06; 95% CI −5.86 to 1.74; *P* = 0.15). There was substantial heterogeneity among berberine combination trials with *I* ^2^ of 82%, *P* < 0.003. So we just performed descriptive analysis where berberine decreased the levels of AST in two trials; and, in one trial, berberine groups substantially reduced the levels of AST from baseline, but the reduction was insignificant compared with control groups (Figure 3).

Four trials reported the data of GGT. These trials involved 397 patients, with 179 patients and 218 patients in treatment groups and control groups, respectively. There was high unexplained heterogeneity of GGT among four trials with *I* ^2^ of 80%, *P* = 0.0005. Berberine decreased GGT in three trials [16–18]; and one trail showed that berberine had a tendency of decreasing GGT in NAFLD patients [21] (Figure 3).

### 3.4. The Effect of Berberine on Blood Glucose in NAFLD Patients

**Figure 4:**
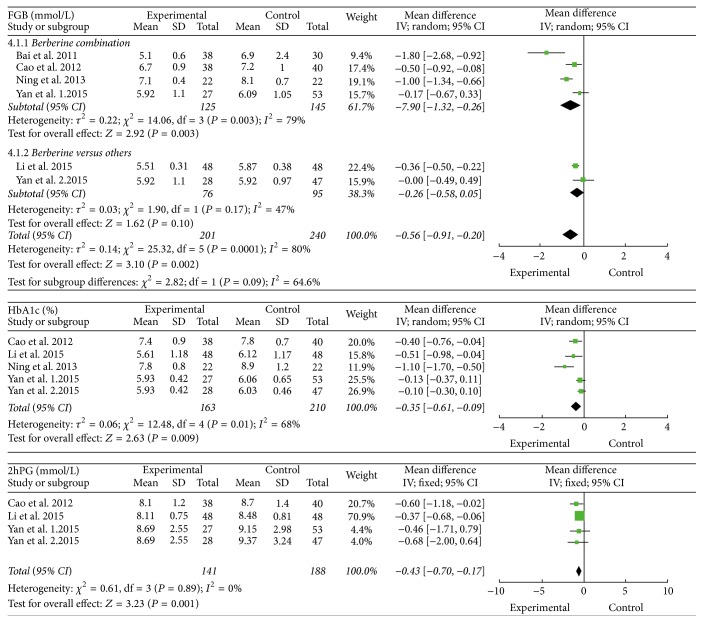
The effect of berberine on blood glucose in NAFLD patients.

Five trials reported the data of FBG. These trials involved 441 patients, with 201 patients and 240 patients in treatment groups and control groups, respectively. High heterogeneity was present in these five studies with *I* ^2^ of 80%, *P* = 0.0001. We preformed the subgroup analysis in the light of berberine-alone groups and berberine combination groups. The analysis indicated that berberine-alone groups were more inclined to reduce the level of FBG in NAFLD patients than other drugs, which were analyzed by a random-effects model (FBG mmol/L: MD = −0.26; 95% CI −0.58 to −0.05; *P* = 0.10). The results of berberine combination for NAFLD patients showed high heterogeneity with *I* ^2^ of 79%, *P* = 0.003. Therefore, description analysis was used and it suggested that the treatment of berberine combination had the tendency of reducing the levels of FBG (Figure 4).

Four trials reported the data of HbA1c. These trials involved 373 patients, with 163 patients and 210 patients in treatment groups and control groups, respectively. There was heterogeneity among these four trials (*I* ^2^ of 68%, *P* = 0.01). A random-effects model analysis was used, and the result showed that berberine reduced HbA1c in NAFLD patients significantly compared with other drugs or lifestyle interventions (HbA1c (%): MD = −0.35; 95% CI −0.61 to −0.09; *P* = 0.01) (Figure 4).

Three trials reported the data of 2hPG. These studies involved 329 patients, with 141 patients and 188 patients in treatment groups and control groups, respectively. There was no significant heterogeneity with *I* ^2^ of 0%, *P* = 0.89. A fixed-effects model analysis showed that berberine significantly reduced 2hPG in NAFLD patients compared with other drugs or lifestyle interventions (2hPG mmol/L: MD = −0.43; 95% CI −0.70 to −0.17; *P* = 0.001) (Figure 4).

### 3.5. The Effect of Berberine on Insulin Resistance in NAFLD Patients

Three trials reported the data of homeostatic model of assessment-insulin resistance (HOMA-IR) using the formula HOMA-IR = FPG × FINS/22.5. These trials involved 301 patients, with 131 patients and 170 patients in treatment groups and control groups, respectively. Among them, one trial providing the data of HOMA-IR with the mean differences and 95% confidence interval cannot undertake the data transformation, and there was high statistical heterogeneity for two other trials with *I* ^2^ of 92%, *P* = 0.0004. Therefore, here we just did descriptive analysis for this indicator.

In the study of Bai et al. 2011 [16] and Yan et al. 2015 [21], compared with lifestyle intervention, berberine could significantly lower HOMA-IR; the study of Cao et al. 2012 [17] found that, compared with single intervention of metformin, metformin combination with berberine could significantly decrease HOMA-IR of patients with NAFLD; Yan et al. 2015 [21] also found that the combination of berberine and pioglitazone had the trend of improving insulin resistance; however, the difference had no statistical significance.

### 3.6. The Effect of Berberine on Fatty Liver in Patients with NAFLD

A total of three trials studied the effect of berberine on fatty liver in patients with NAFLD, involving a total of 259 patients, and the treatment group consisted of 107 patients and the control group consisted of 152 patients. There were 2 trials [19, 20] that used the liver ultrasonic examination to evaluate the degree of fatty liver, and 1 trial [21] evaluated the degree of fatty liver by a proton magnetic resonance spectroscopy (H MRS). Due to different evaluation methods, the data cannot be merged, and we just did descriptive analysis.

In the study of Yan et al. 2015 [21], it was found that, compared with lifestyle intervention group, berberine could significantly lower liver lipid content in patients with NAFLD; in the study of Xie et al. 2011 [20], it was found that the obvious effect rate was 70% after treatment with berberine via liver type-B ultrasonic examination, and there was no statistically significant difference compared with control group; in the study of Ning et al. 2013 [19] with liver type-B ultrasonic examination hemodynamic evaluation of fatty liver found that, compared with control group, berberine could significantly improve the condition of fatty liver in patients with NAFLD.

## 4. Discussion

Currently, the incidence of NAFLD shows an increased and low aging tendency along with the improvement of people's living standard, and, in patients with type 2 diabetes and obesity, the incidence of NAFLD is much higher. Studies found that, compared with normal people, patients with NAFLD had a higher mortality rate, and the risk for the development of cardiovascular disease and metabolic syndrome related cancer increased [22]. Nowadays, researches of NAFLD receive more attention, and NAFLD has become one of the world's important public health problems in the 21st century.

It is still important that improving the way of life and exercise dominate the treatment for NAFLD; however, patients often cannot adhere to it because the effect needs a long term to be seen, so adherence to this therapy is poor. Other treatments for NAFLD include drug therapy (lipid-lowering drugs, insulin sensitizing agent, liver-protection medicine, antioxidant, etc.) and surgery, but their efficacies are not precise.

With meta-analysis through 6 studies including 501 patients, this review suggests that berberine has a positive effect on many aspects in patients with NAFLD including improving blood lipids (TC, TG, and LDL), liver function (ALT, AST), and blood glucose (FBG, 2hPG, and HbA1c). Compared with control group, berberine can decrease the TC, LDL, ALT, 2hPG, and HbA1c level in patients with NAFLD, and the difference is statistically significant. The subgroup analysis of studies on TG, AST, and FBG indicates that the combination of berberine treatment can significantly reduce TG levels in patients with NAFLD, and results of researches on combination of berberine treatment tend to the fact that berberine has the effect of reducing AST and FBG, but, at present, the results have considerable heterogeneity. Compared with other drugs, treatment with berberine alone reduced TG levels in patients with NAFLD, and the effect of berberine alone has a tendency to decrease the AST and FBG in patients with NAFLD. Berberine also has an improvement effect on NAFLD patients' insulin resistance and fatty liver condition.

Since the number of included studies was fewer than 10, this review did not assess risk of publication bias. The research time of these articles included in this study was shorter, in which the longest one was 16 weeks, while NAFLD is chronic disease, and it might take longer time to manage the improvement of liver pathological index and biochemical index. Therefore, it cannot well figure out the therapeutic effect of berberine on NAFLD, which might be shown better if more studies with longer term were included. In addition, articles included in this study did not provide the methods of blind and allocation concealment, and the random allocation concealments of most studies were not described in detail. In terms of evaluation indexes, not widely using imaging evaluation including CT and MRI, besides the small sample sizes of most articles included, can be the factors causing bias; therefore we should be cautious about the results of the meta-analysis.

In conclusion, based on current evidence, berberine can significantly improve blood lipids and liver function in patients with NAFLD and has good advantage in reducing blood glucose in patients with NAFLD, which might be a new choice for the treatment of NAFLD. Due to the limit of the number and quality of the trials included, the conclusions need to be further validated by more strictly designed multicentered RCTs of high quality and large scale.

## Figures and Tables

**Table 1:** Characteristics of the 6 included studies.

Study	Sample size, *n*	Intervention of experimental group	Intervention of control group	Dose of berberine	Duration	Outcomes
Bai et al. 2011 [16]	78	BBR	ISI	0.5 g, tid	3 months	1, 2, 3, 5, 6, 7, 8, 11
Cao et al. 2012 [17]	78	BBR + metformin	Metformin	0.5 g, tid	16 weeks	1, 2, 3, 4, 5, 6, 7, 8, 9, 10, 11
Li 2015 [18]	96	BBR	Acarbose	0.3 g, tid	3 months	1, 2, 3, 4, 5, 6, 7, 8, 9, 10
Ning et al. 2013 [19]	44	BBR + metformin	Metformin	0.5 g, tid	16 weeks	1, 2, 8, 10
Xie et al. 2011 [20]	60	BBR	Xuezhikang	0.3 g, tid	12 weeks	1, 2, 3, 4, 5, 6
Yan et al. 2015 [21]	155	BBR	ISI or pioglitazone	0.5 g, tid	16 weeks	1, 2, 3, 4, 5, 6, 7, 8, 9, 10, 11

1: TC; 2: TG; 3: LDL; 4: HDL; 5: ALT; 6: AST; 7: GGT; 8: FBG; 9: 2hPG; 10: HbA1c; 11: HOMA-IR.

**Table 2:** Risk of bias assessment.

Type of bias	Bai et al. 2011 [16]	Cao et al. 2012 [17]	Li 2015 [18]	Ning et al. 2013 [19]	Xie et al. 2011 [20]	Yan et al. 2015 [21]
Random sequence generation (selection bias)	?	?	Low	?	?	Low
Allocation concealment (selection bias)	?	?	High	?	?	?
Blinding of participants and personnel (performance bias)	?	?	?	?	?	?
Blinding of outcome assessment (detection bias)	?	Low	Low	Low	Low	Low
Incomplete outcome data (attrition bias)	Low	?	Low	Low	Low	High
Selective reporting (reporting bias)	Low	Low	Low	?	Low	Low
Other biases	Low	Low	Low	Low	Low	Low
